# Supplementary material for: The cascade of global trade to large climate forcing over the Tibetan Plateau glaciers
Source: Nat Commun. 2019 Jul 23;10:3281. doi: 10.1038/s41467-019-10876-9 (PMC6650455; doi:10.1038/s41467-019-10876-9)
Supplement: Supplementary file 1 — Supplementary Information [file 41467_2019_10876_MOESM1_ESM.pdf]

Supplementary Information

The Cascade of Global Trade to Large Climate Forcing over the Tibetan Plateau Glaciers

Yi et al.

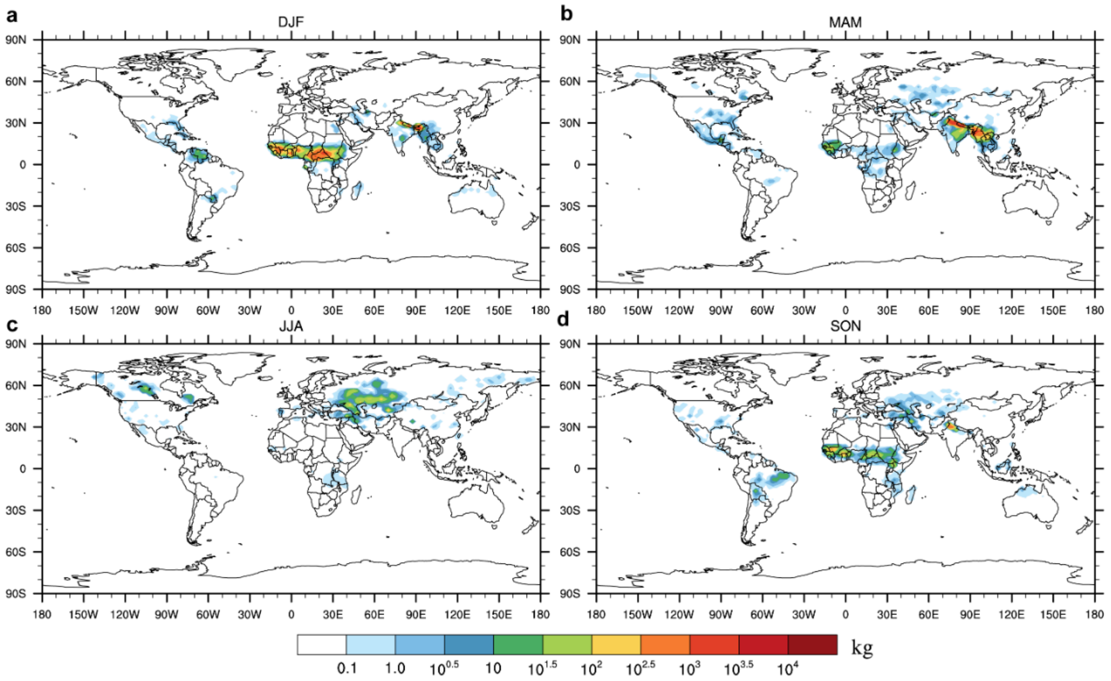

Supplementary Figure 1. Global biomass burning contributions to BC over the HTP glaciers. The spatial distributions of global biomass burning BC sources contributed to the BC mass-burden over the selected HTP glacier regions (defined in Fig. 1) during DJF (December-January-February, a), MAM (March-April-May, b), JJA (June-July-August, c), and SON (September-October-November, d) in 2011 from the adjoint simulations.

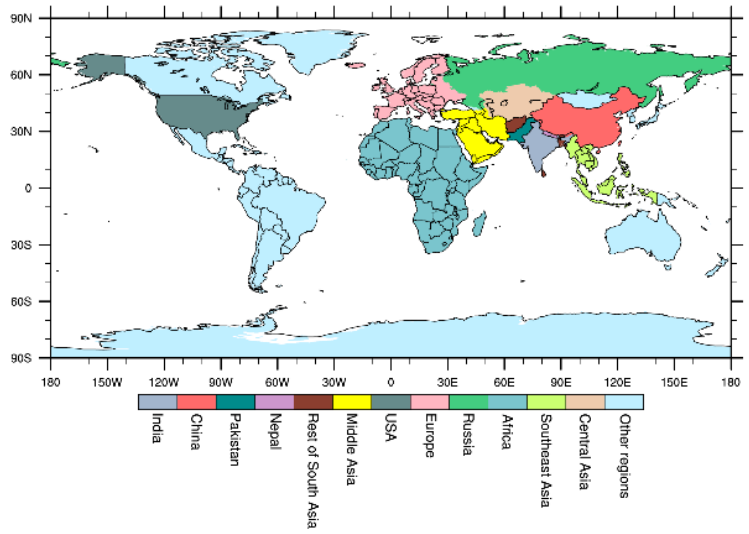

Supplementary Figure 2. Definition of the 13 world regions used in this study.

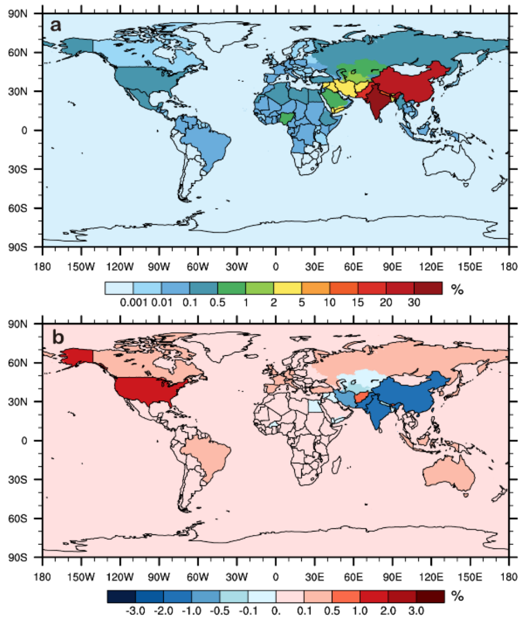

Supplementary Figure 3. Relative contributions from different countries/regions to the BC over the HTP glaciers from production and consumption perspectives. a, Relative contributions from different countries/regions to the BC over the HTP glaciers according to the production-based accounting method. b, Differences in the results from a consumption perspective relative to those from a production perspective.

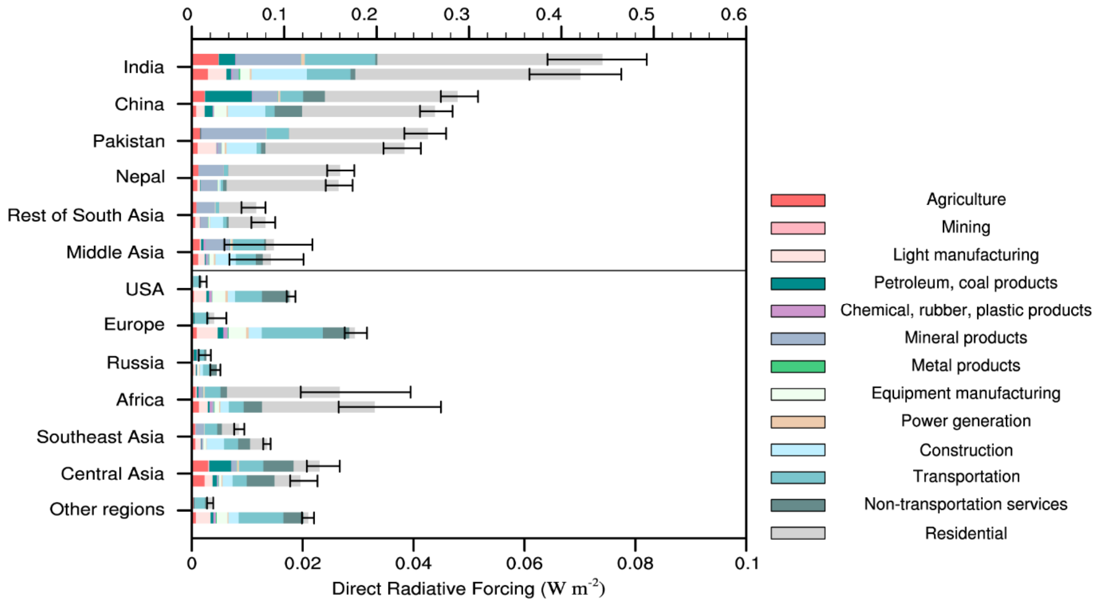

Supplementary Figure 4. Black Carbon direct radiative forcing over the HTP. Production- (top bar), consumption-based (bottom bar) direct radiative forcing at the top-of-atmosphere of BC averaged over the HTP glaciers for different regions and sectors. The results for India, China, Pakistan, Nepal, Middle Asia, and the rest of South Asia are displayed according to the x-axis in the top half. The results for the USA, Europe, Russia, Africa, Southeast Asia, Central Asia, and all other regions are displayed according to the x-axis on the bottom half. Error bars denote uncertainty ranges related to inter-annual climate variabilities.

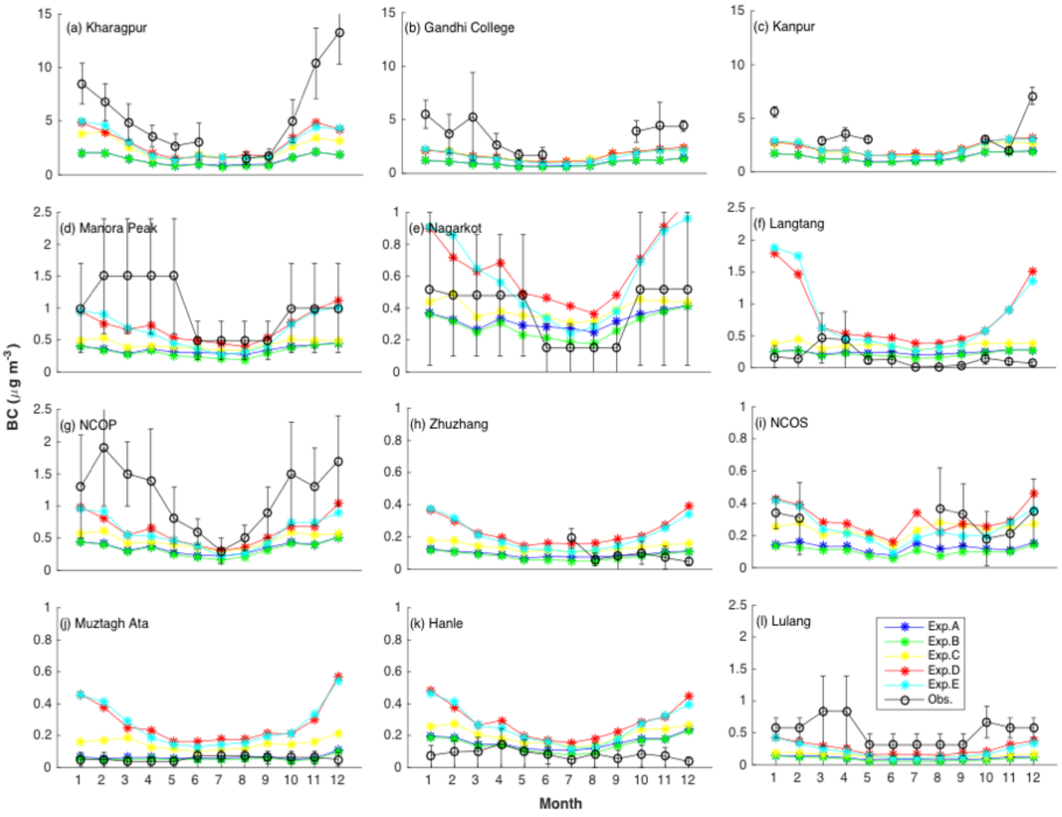

Supplementary Figure 5. Observed (black curve) and simulated (coloured curves) monthly mean surface concentrations of BC over the HTP and adjacent regions. Twelve measurement sites are used in this study: Kharagpur (a), Kanpur (b), Gandhi College (c), Nagarkot (d), Langtang (e), Nepal Climate Observatory at Pyramid (NCOP) (f), Manora Peak (g), Nam Co Observational Station (NCOS) (h), Zhuzhang (i), Muztagh Ata (j), Hanle (k) and Lutang (l). Only seasonal mean observations are available at sites d, e, and l. Error bars represent the standard deviations of the observations at all sites except for site k. For site k, the 5 and 95th percentile values are shown as the ends of the error bars instead. See Supplementary Table 2 for the details of these sites.

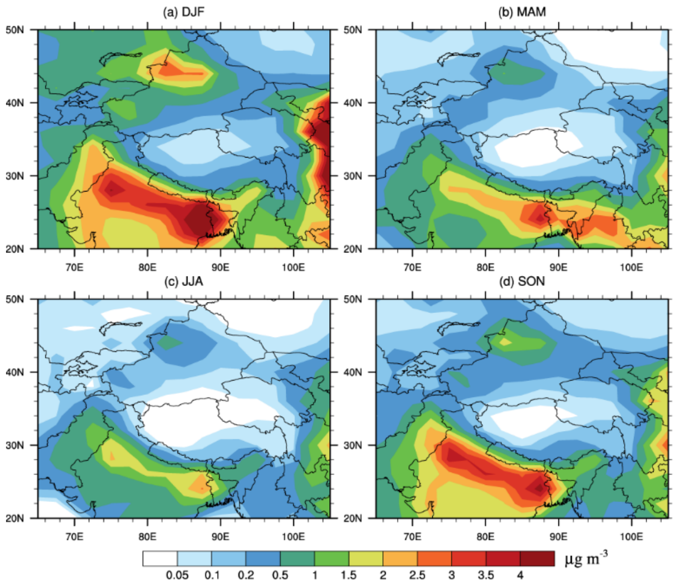

Supplementary Figure 6. Simulated seasonal-mean BC surface concentrations over the HTP and adjacent regions.

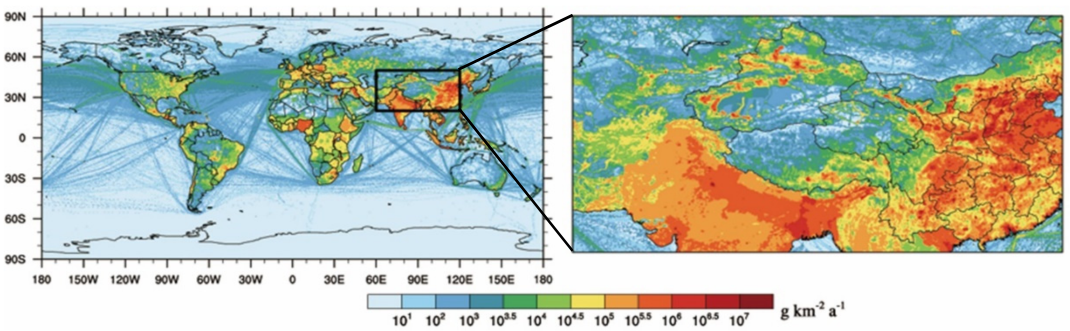

Supplementary Figure 7. Distributions of BC anthropogenic emission in 2011. The BC emission data set with a 0.1° × 0.1° resolution is obtained from the PKU-BC-Inventory[1].

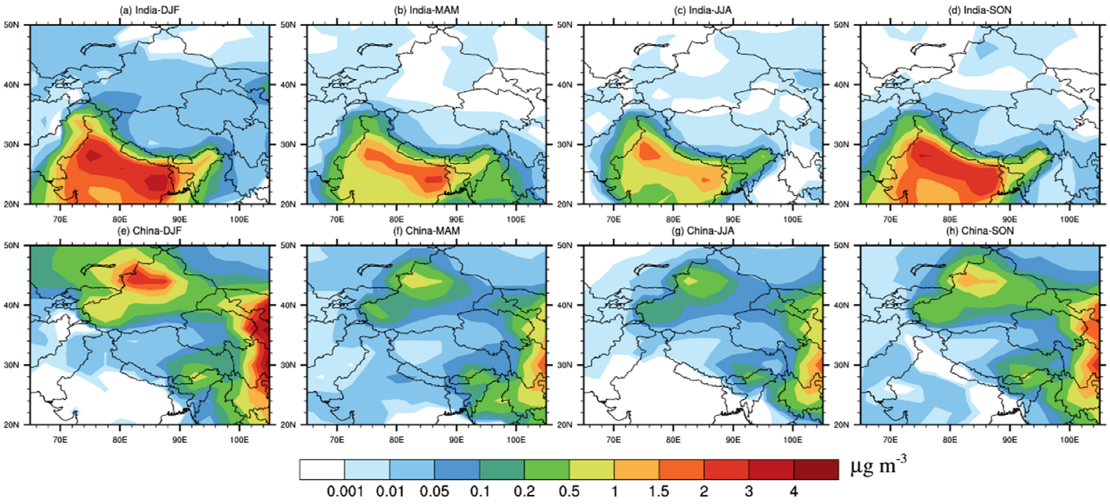

Supplementary Figure 8. The seasonal-mean BC surface concentrations over the HTP and adjacent regions contributed by consumption-based emissions from India (a-d) and China (e-h). These results are estimated using the GEOS-Chem forward model simulations. We use the GEOS-Chem forward model to conduct a base simulation in which all BC emissions are included and two sensitivity simulations in which the consumption-based BC emissions from China and India are removed. Differences in BC surface concentrations between the base simulation and sensitivity simulations are showed here.

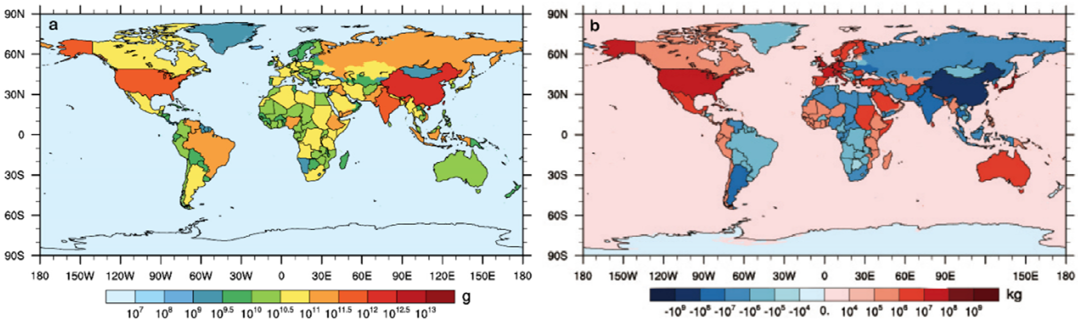

Supplementary Figure 9. Production- and consumption-based BC emissions of different countries/regions in 2011. a, Annual total BC emissions from different countries/regions according to the production-based accounting method. b, Differences in consumption-based BC emissions relative to the production-based emissions.

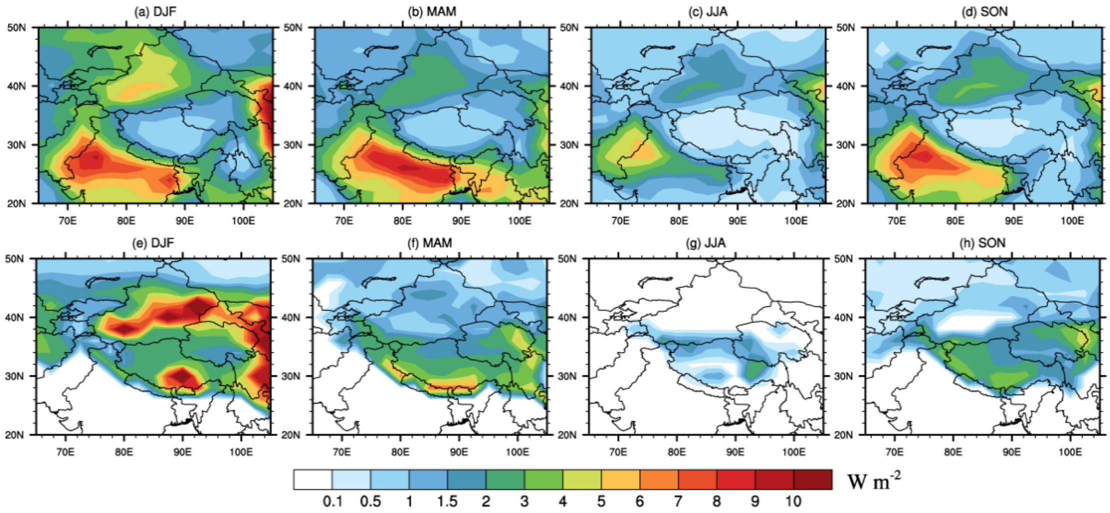

Supplementary Figure 10. Simulated seasonal mean BC radiative forcing. a-d, Simulated BC direct radiative forcing (DRF) at the top-of-atmosphere (TOA) over the HTP and adjacent regions during different seasons. e-h, Simulated BC snow albedo forcing (SAF) over the HTP and adjacent regions during different seasons.

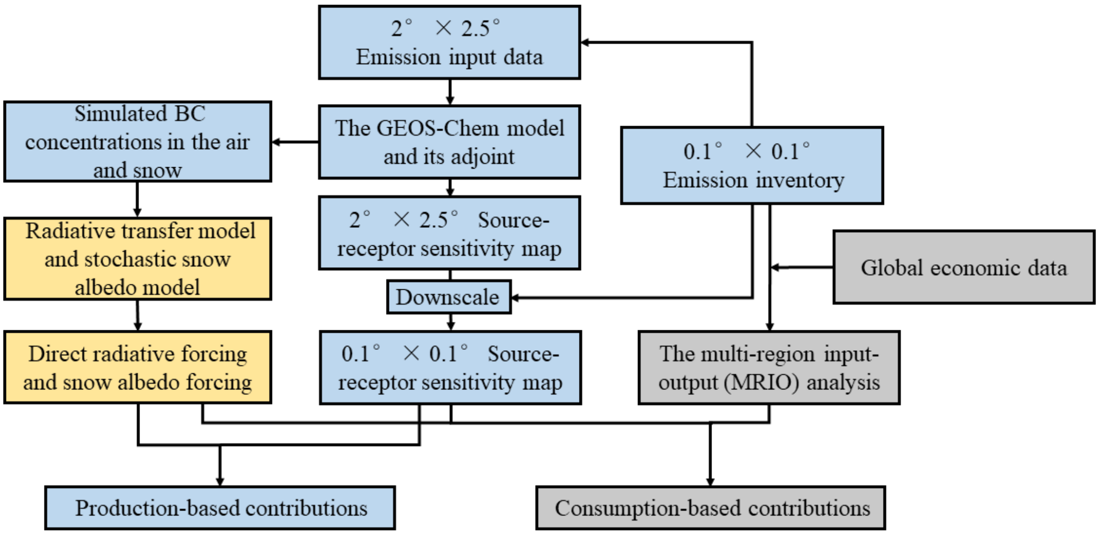

Supplementary Figure 11. Schematic methodology for the combined adjoint and multi-region input-output analysis.

Supplementary Table 1. List of GEOS-Chem model experiments

Experiment	A	B	C	D	E
Anthropogenic emissions	Bond et al. (2007)	Bond et al. (2007)	China and India: Lu et al. (2011)<br>Rest of Asia: Zhang et al. (2009)<br>Rest of world: Bond et al. (2007)	Wang et al. (2014)	Wang et al. (2014)
Biomass burning emissions	GFEDv3 (van der Werf et al., 2010)				
BC ageing	e-folding time 1.15 days	He et al. (2016)	e-folding time 1.15 days	e-folding time 1.15 days	He et al. (2016)

Supplementary Table 2. List of measurement sites of surface BC concentrations used in this study for the model evaluation

Site	Lat(°N)	Long（°E)	Elev.(m)	Time	Technique	Source
Nainital	29.2	79.3	1950	2006 Jan-May	Aethalometer	Beegum et al.(2009)
Kharagpur	22.5	87.5	28	2005-2007	Aethalometer	Nair et al.(2012)
Kanpur	26.5	80.3	142	2007-2008	TOT	Ram et al.(2010b)
Gandhi College	25.9	84.1	158	Sep.-Dec. 2006	retrieval	Ganguly et al.(2009b)
Nagarkot	27.7	85.5	2150	1999-2000	TOT	Carrico et al.(2003)
Langtang	28.1	85.6	3920	1999-2000	TOT	
NCOP	28.0	86.8	5079	2006-2008	MAAP	Bonasoni et al.(2010)
Manora Peak	29.4	79.5	1950	2005-2008	TOT	Ram et al.(2010a)
NCOS	30.8	91.0	4730	2006	TOR	Ming et al.(2010)
				2012	TOR	Wan et al.(2015)
Zhuzhang	28.0	99.7	3583	Aug.2004-Feb.2005	TOR	Qu et al.(2008)
Muztagh Ata	38.3	75.0	4500	2003-2006	TOR	Cao et al. (2009)
Hanle	32.8	79.0	4250	2009-2010	Aethalometer	Babu et al. (2011)
Lulang	29.5	94.4	3300	2008-2009	TOR	Zhao et al. (2013)
QOMS	28.4	87.0	4276	Aug. 2009-Jul. 2010	TOR	Cong et al.(2015)
				May 2015-May 2017	Aethalometer	Chen et al.(2018)
Beiluhe	34.9	92.9	4600	Nov. 2012-Jun. 2013	Aethalometer	Wang et al.(2016)
Ranwu	29.3	97.0	4600	Nov. 2012-Jun. 2013	Aethalometer	
QSSGEE	39.5	96.5	4214	May 2009-Mar. 2011	Aethalometer	Zhao et al.(2012)

*TOT: thermal-optical transmittance; MAAP: multi-angle absorption photometer; TOR thermal-optical reflectance.

Supplementary Table 3. List of measurement sites of the BC concentration in snow used in this study for the model evaluation

Site	Lat(°N)	Long（°E)	Elev.(km)	Time	Source
Zuoqiupu	29.21	96.92	5.5	monsoon 2006	Xu et al.(2009)
	29.21	96.92	5.6	non-monsoon 2006	
Qiangyong	28.83	90.25	5.4	summer 2001	Xu et al.(2006)
Noijin Kangsang	29.04	90.2	5.95	annual 2005	Xu et al.(2009)
East Rongbuk	28.02	86.96	6.5	monsoon 2001	Ming et al.(2008)
	28.02	86.96	6.5	non-monsoon 2001	
	28.02	86.96	6.5	summer 2002	Ming et al.(2009a)
	28.02	86.96	6.5	Oct.2004	
	28.02	86.96	6.5	Sept.2006	Ming et al.(2013)
	28.02	86.96	6.52	May 2007	Ming et al.(2012)
Kangwure	28.47	85.82	6	summer 2001	Xu et al.(2006)
Namunani	30.45	81.27	5.9	summer 2004	
Mt.Muztagh	38.28	75.02	6.35	summer 2001	
	38.28	75.1	6.3	1999	
Laohugou #12	39.43	96.56	5.05	Oct.2005	Ming et al.(2009a)
Qiyi	39.23	97.06	4.85	Jul.2005	Ming et al.(2010）
1 July glacier	39.23	97.75	4.6	summer 2001	Xu et al.(2006)
Meikuang	35.67	94.18	5.2	Nov.2005	Ming et al.(2009b)
Dongkemadi	33.1	92.08		summer 2001	Xu et al.(2006)
	33.1	92.08	5.6	year 2005	Ming et al.(2013)
	33.1	92.08		Aug.2014-Oct.2015	Li et al.(2017)
La'nong	30.42	90.57	5.85	Jun.2005	Ming et al.(2009a)
Zhadang	30.47	90.5	5.5-5.8	Jul.2006	Ming et al.(2010)
	30.47	90.5	5.8	Jul.-Aug. 2012	Qu et al.(2014)
Haxilegen River	43.73	84.46	3.76	Oct.2006	Ming et al.(2011a)
Urumqi Riverhead	43.1	86.82	4.05	Nov.2006	Ming et al.(2009b)
Miao'ergou #3	43.06	94.32	4.51	Aug.2005	Ming et al.(2009a)
Demula glacier	29.35	97.2	5.1	Jun.2015	Zhang et al.(2017)
Muji glacier	39.19	73.74	5.5	Jul.2012	Yang et al.(2015)
Tianshan Urumpi glacier #1	43.1	86.82	3.9	Aug. 2013	Ming et al.(2016)

Supplementary Table 4. Observed and simulated surface BC concentrations over the Himalayas and the Tibetan Plateau (HTP) and adjacent regions.

Site	Time	BC in surface air (μg m^{-3})					
		Obs.*	Exp.A	Exp.B	Exp.C	Exp.D	Exp.E
Nainital	2006 Jan-May	1.4[a]	0.50	0.49	0.71	0.93	0.92
Kharagpur	2005-2007	6.2±4[b]	1.42	1.37	2.43	2.90	2.85
Kanpur	2007-2008	3.8±2.3[c]	0.99	0.96	1.71	1.68	1.56
Gandhi College	Sep.-Dec. 2006	4.75±0.48[d]	1.78	1.75	2.58	2.82	2.71
Nagarkot	1999-2000	1.0±0.63[e]	0.35	0.31	0.43	0.69	0.64
Langtang	1999-2000	0.38±0.34[e]	0.32	0.29	0.39	0.65	0.60
NCOP	2006-2008	0.15±0.14[f]	0.24	0.22	0.36	0.80	0.77
Manora Peak	2005-2008	1.14±0.55[g]	0.35	0.33	0.46	0.62	0.59
NCOS	2006	0.08±0.07[h]	0.09	0.08	0.14	0.23	0.21
	2012	0.19±0.11[i]	0.09	0.08	0.14	0.23	0.21
Zhuzhang	Aug.2004-Feb.2005	0.34±0.18[j]	0.13	0.11	0.26	0.33	0.29
Muztagh Ata	2003-2006	0.06±0.04[k]	0.15	0.05	0.06	0.27	0.27
Hanle	2009-2010	0.08±0.06[l]	0.06	0.04	0.05	0.08	0.07
Lulang	2008-2009	0.52±0.35[m]	0.06	0.03	0.05	0.14	0.11
QOMS	Aug. 2009-Jul. 2010	0.25±0.2[2n]	0.20	0.17	0.29	0.73	0.71
	May 2015-May 2017	0.3±0.34[o]	0.20	0.17	0.29	0.73	0.71
Beiluhe	Nov. 2012-Jun. 2013	0.4[p]	0.05	0.04	0.07	0.11	0.10
Ranwu	Nov. 2012-Jun. 2013	0.14[p]	0.12	0.10	0.18	0.30	0.26
QSSGEE	May 2009-Mar. 2011	0.1[q]	0.09	0.08	0.16	0.28	0.24

* Multi-month averaged values and standard deviations are provided for different measurement sites. Sources: [a]Beegum et al.(2009), [b]Nair et al. (2012), [c]Ram et al. (2010b), [d]Ganguly et al. (2009), [e]Carrico et al. (2003), fBonasoni et al. (2010), [g]Ram et al. (2010a), [h]Ming et al. (2010), [i]Wan et al.(2015), [j]Qu et al. (2008), [k]Cao et al. (2009), [l]Babu et al. (2011), [m]Zhao et al. (2013),[n]Cong et al.(2015), [o]Chen et al.(2018), [p]Wang et al.(2016), [q]Zhao et al.(2012).

Supplementary Table 5. Statistical errors in the 2006 simulated BC concentrations in the surface air and snow over the HTP and adjacent regions.

Statistical errors	Exp. A	Exp. B	Exp. C	Exp. D	Exp. E
	BC in surface air (μg m^{-3})				
Mean error	-0.74	-0.77	-0.55	-0.35	-0.39
Mean absolute error	0.76	0.77	0.60	0.63	0.64
Root-mean-square error (RMSE)	1.48	1.50	1.15	1.05	1.08
	BC in snow (μg kg^{-1})				
Mean error	-11.94	-16.88	-6.72	-7.88	-7.35
Mean absolute error	27.34	28.88	23.28	26.20	25.33
Root-mean-square error (RMSE)	37.05	40.01	29.96	35.12	33.49

Supplementary Table 6. Observed and simulated BC concentrations in snow over the HTP and adjacent regions

Site	Time	BC in snow (μg kg^{-1})					
		Obs.	Exp.A	Exp.B	Exp.C	Exp.D	Exp.E
Zuoqiupu	Monsoon 2006[a]	7.9	21.01	11.42	30.43	38.08	19.53
	Non-monsoon 2006[a]	15.9	42.81	37.64	49.67	76.84	48.77
Qiangyong	Summer 2001[b]	43.1	27.18	23.49	41.23	26.30	18.77
Noijin Kangsang	Annual 2005[a]	30.6	41.05	26.15	52.65	31.58	24.49
East Rongbuk	Monsoon 2001[c]	35	30.64	30.72	24.15	25.32	17.10
	Non-monsoon 2001[c]	21	60.03	57.09	28.85	43.78	26.33
	Summer 2002[c]	20.3	30.31	30.96	24.40	24.08	16.41
	Oct.2004[d]	18	39.27	38.98	22.33	24.15	26.00
	Sept.2006[e]	9	31.62	30.02	23.38	29.05	19.17
	May 2007[f]	41.8	35.22	36.79	25.94	21.45	17.61
Kangwure	Summer 2001[b]	21.8	20.16	15.24	21.45	22.66	13.88
Namunani	Summer 2004[b]	4.3	10.72	10.72	10.72	10.72	10.72
Mt.Muztagh	Summer 2001[b]	37.2	25.60	23.54	27.54	43.52	36.70
	1999[b]	26.6	22.23	17.59	38.59	76.92	47.82
Laohugou #12	Oct.2005[d]	35	34.88	35.49	50.25	31.63	55.24
Qiyi	Jul.2005[g]	22	35.54	24.26	35.23	49.07	31.95
1 July glacier	Summer 2001[b]	52.6	25.98	19.87	24.91	30.06	24.31
Meikuang	Nov.2005[h]	81	11.83	8.97	15.64	29.78	22.13
Dongkemadi	Summer 2001[b]	18.2	12.09	5.23	16.91	21.28	12.34
	2005[e]	36	12.96	6.58	14.50	25.05	16.83
	Aug.2014-Oct.2015[i]	41.8	11.17	5.89	15.48	20.33	13.33
La'nong	Jun.2005[e]	67	16.45	7.61	27.25	19.43	19.17
Zhadang	Jul.2006[g]	87.4	14.67	6.18	23.98	31.72	16.09
	Jul.-Aug. 2012[j]	91.8	16.60	6.80	24.57	28.55	16.02
Haxilegen River	Oct.2006[k]	46.9	52.85	50.80	74.72	39.73	119.66
Urumqi Riverhead	Nov.2006[h]	141	39.13	38.22	98.20	51.20	146.67
Miao'ergou #3	Aug.2005[d]	111	63.05	47.37	86.59	44.48	80.77
Demula glacier	Jun.2015[l]	56.6±26.1	15.83	7.80	24.93	31.64	16.94
Muji glacier	Jul.2012[m]	25	63.23	58.32	36.98	56.30	34.46
Tianshan Urumpi glacier #1	Aug. 2013[n]	42.5	66.01	62.10	95.09	47.12	98.48

Sources: [a]Xu et al. (2009), bXu et al.(2006),cMing et al.(2008), [d]Ming et al. (2009a), [e]Ming et al. (2013), [f]Ming et al. (2012), [g]Ming et al. (2010), [h]Ming et al. (2009b), [i]Li et al.(2017), [j]Qu et al.(2014), [k]Ming et al.(2011a),[l]Zhang et al.(2016), [m]Yang et al.(2015), [n]Ming et al.(2016).

Supplementary Discussion

Model evaluation

Emission inventories and air transport patterns are the two major factors that determine the performance of a global CTM. Here we conduct five experiments composed of different emission inventories and BC ageing schemes to obtain the optical model configuration for the adjoint simulation (Supplementary Table 1). The biomass burning emissions in all experiments are derived from the Global Fire Emissions Database version 3 (GFED3) [2]. The default anthropogenic BC emission inventory employed within GEOS-Chem is from Bond et al.[3]. Their estimation revealed a total global anthropogenic BC emission mass of 4.4 Tg for the year 2000 [3]. However, coincident with its rapid economic growth and industrial expansion, the BC emissions from Asia changed significantly after 2000 [4]. Therefore, over the past two decades, the BC emission inventory for Asia has undergone a series of updates using the latest available economic data and emission factors [4-6]. He et al. [7] showed that the use of updated anthropogenic BC emission inventories from Lu et al. [4] for China and India and from Zhang et al. [5] for the rest of Asia considerably improves the BC simulation model performance over the HTP. Wang et al. [1] further developed a new global BC emission inventory by using recompiled fuel consumption and emission factor data sets (PKU-BC-Inventory, http://inventory.pku.edu.cn/). Their inventory was evaluated with regard to the modeling of ambient surface BC concentrations, and it demonstrated a better agreement with field observations than previous inventories [1].

In addition to emission inventories, uncertainties in the BC ageing process (i.e., the transformation of BC from hydrophobic to hydrophilic) also contribute to biases between model simulations and observations [8,9]. In the GEOS-Chem model, the ageing process is not explicitly simulated but rather parameterized simply through a fixed e-folding time of 1.15 days [10]. Recently, He et al. [11] developed a microphysics-based BC ageing scheme in GEOS-Chem by explicitly computing the condensation of soluble materials onto hydrophobic BC and the coagulation between hydrophobic BC and pre-existing hydrophilic particles.

The model experiments are evaluated using in situ BC measurements of the surface air and snow over the HTP and adjacent regions. Here, we choose 17 sites with monthly or seasonal measurements of the surface BC concentration [12-27] and 20 measurement sites with measurements of the BC concentration in snow [17,28-38] for a comparison with the model results. The locations of these sites are shown in Fig. 1, and their detailed descriptions are provided in Supplementary Table 2 and 3, including the locations and elevations of the sites, the sampling time periods and the observation methods.

The observed and simulated surface BC concentrations in the HTP are shown in Supplementary Table 4 and Supplementary Fig. 5. The simulated BC concentrations at specified sites were obtained using a simple inverse distance weighted interpolation from neighbouring model grids to compare with observations. The modelling results

using the Bond, et al. [3] emissions (i.e., Exp. A and Exp. B) significantly underestimate the surface BC concentrations at all measurement sites except for NCOP and Muztagh Ata. In Exp. C, these negative biases are partly improved by replacing the Bond et al. [3] emissions with the Lu et al. [4] inventory for China and India and with the Zhang et al. [5] inventory for the rest of Asia; this suggests that the Bond et al. [15] inventory underestimates the BC emissions in Asia, although it still underestimates the surface BC concentrations, especially for polluted regions with high observation values (see Supplementary Table 4). Experiments using the Wang et al. [1] inventory (i.e., Exp. D and Exp. E) successfully reproduce the observed BC concentrations at most sites except for NCOP, Manora Peak, Muztagh Ata, Lulang, QOMS, and Beiluhe. These experiments overestimate the annual mean surface BC concentrations at NCOP, QOMS, and Muztagh Ata and underestimate those at Manora Peak, Lulang, and Beiluhe by a factor ranging from 2 to 4 with no systematic bias. Accordingly, since the modelling results represent the mean concentration over a large area while the in situ measurements are acquired at a small sampling point, large discrepancies at these measurement sites may be observed between the modelled and observed values. Statistical analysis (i.e., of the mean errors, mean absolute errors and root-mean-square errors) reveals that the modelling results in Exp. D and Exp. E are in better agreement with the observations than those in the other experiments, which have lower statistical errors (Supplementary Table 5).

The simulated BC concentrations in snow are calculated as the ratio of the total deposited BC to the total precipitation following a previous approach [7], and they are compared with the in situ measurements (Supplementary Table 6). Note that this approach approximates the BC concentration in snow while neglecting the effects of snow ageing and the fractions of rain within the total precipitation, and thus, this technique may introduce some uncertainties [7]. Here, we define June-September as the monsoon season and October-May as the non-monsoon season following Xu et al. [28]. Supplementary Table 6 shows that all of the experiments overestimate the BC concentrations in snow by a factor from 2 to 4 at the Zuoqiupu, Namunani, Qiyi, Haxilegen River, Muji glacier, and Tianshan Urumpi glacier #1 sites while underestimate the results at Qiangyong, 1 July glacier, Meikuang, Dongkemadi, and Demula glacier sites. The modelling results using updated emission inventories (i.e., Exp. C, Exp. D and Exp. E) improve the model performances in underestimations for most sites. Meanwhile, they also produce higher BC concentrations in snow at Mt. Muztagh and Urumqi Riverhead. It is likely that the underestimations in our simulation results at most sites can be mainly attributed to the lack of considering the snow aging and BC post-depositional process in our calculations [7,39]. He et al. [7] further indicated that these overestimations are likely caused by excessive BC deposition. The BC ageing process is an important factor for determining the scavenging efficiency of BC during the modelling process. Compared with the experiments utilizing a fixed e-folding time for the BC ageing approximation, the experiments using the microphysics-based BC ageing scheme estimate lower BC concentrations in the atmosphere and snow (Supplementary Table 4 and 6). He et al. [11] showed that the microphysics-based BC ageing scheme has a higher BC ageing rate over source regions than a fixed ageing

scheme with an e-folding time and therefore considerably reduces the model bias in remote regions. In this study, we find that Exp. E, which employs the microphysics-based BC ageing scheme, has lower statistical errors during the simulation of the BC concentrations in snow than Exp. D (Supplementary Table 5).

Overall, comparisons with available observations demonstrate that the GEOS-Chem model exhibits a good performance in the simulation of the atmospheric transport and deposition of BC over the HTP. Generally, the model captures the seasonal and spatial variations in the BC concentrations in the surface air and snow, although it may under- or overestimate the BC concentrations at individual sites. The simulated BC concentrations in the surface air and snow show statistically better agreement with the in situ observations when using the updated BC emission inventory from PKU-BC-Inventory [1] and the microphysics-based BC ageing scheme from He et al. [11] in comparison with other cases. The remaining discrepancies between the model results and observations can be partly attributed to the inherent difficulties in representing the complex topography and meteorological field over the HTP due to the relatively coarse resolution of the global model. We hence choose the BC ageing scheme from He et al. [11] and the anthropogenic BC emission inventory from PKU-BC-Inventory [1] for our adjoint simulations.

Uncertainties and limitations

The uncertainties and limitations in our results are derived from several factors that are discussed below.

The uncertainties in the anthropogenic BC emissions (i.e., production-based emissions) are substantial; they are largely due to a lack of knowledge regarding activity data, technology splits, and emission factors. The default anthropogenic BC emission inventory from Bond et al. shows a 95 % uncertainty range that can reach a factor of four [3]. Using a Monte Carlo method, the 95 % uncertainties in the BC emission inventory from Lu et al.[4] are estimated to range from -43 to 90 % for China and from -41 to 87 % for India. For the PKU-BC-Inventory, a Monte Carlo simulation yields an interquartile range (IQR) from -40 to 70 % relative to the mean value [1]. Generally, these uncertainties are larger in developing countries where pertinent information, including activity rates and emission characteristics, are scarcer than in developed countries [40]. The GFEDv3 emissions used in our simulations for the biomass burning sources of BC also display an uncertainty of at least 20 % globally and higher in boreal regions and equatorial Asia; these uncertainties are mainly related to insufficient data regarding burned area, fuel loads, and emission factors [2].

The uncertainties in the production-based BC emissions are further propagated into the estimations of consumption-based emissions during the assigning of emissions produced in one region to final consumers and primary suppliers throughout the global trade. In addition, the MRIO model introduces uncertainties into the consumption-based emissions associated with inaccuracies in economic statistics and sector aggregations. Peters et al. [41] estimated the variations in consumption-based carbon

emissions calculated using five different MRIO models and indicated that the uncertainties related to the MRIO calculations are much smaller than those in production-based inventories. Previous studies generally assumed that uncertainties related to MRIO analysis are ~10 % relative to the uncertainties in production-based emissions [42,43].

In addition to emission inventories, the simulated BC concentrations in the surface air and snow are further determined through model representations of chemical and physical processes. A model evaluation shows that the simulated BC concentrations in the surface air fall within the uncertainty range of observations at most measurement sites and agree with the observations within a factor of two (Supplementary Table 4). Uncertainties in the simulated BC concentrations in snow yield a factor range of 2-4 compared with the observations (Supplementary Table 6). The BC simulation model performance is inherently related to the emission inventories employed and substantially relies on the BC ageing and scavenging processes. The calculation of the BC concentrations in snow introduces a large component of the overall uncertainty since we neglect the effects of snow ageing and the fractions of rain within the total precipitation during our estimation. Biases in the GEOS-5 precipitation fields also contribute to uncertainties in the simulated BC concentrations in snow. Caution should therefore be taken while using the deviations between the simulated and observed BC concentrations to represent the overall model errors because of the existence of systemic errors within the observations and because of representative errors when comparing localized observations with modelling results for a large area.

The adjoint analysis not only shares the same errors with the GEOS-Chem forward simulations but also introduces additional uncertainties when computing the sensitivity of BC concentrations in the receptor box to global emissions. Qi et al. [44] compared the performance of the adjoint model with the forward model during a sensitivity analysis and found that the disagreement is within ~15 %. We conduct two sensitivity simulations using the GEOS-Chem forward model in which the consumption-based BC emissions from China and India are removed. Supplementary Fig. 8 shows the corresponding changes in the BC surface concentrations over the HTP and adjacent regions. The results reveal that removing the consumption-based BC emissions of China and India will reduce the BC column concentrations over the HTP glaciers by 19.2 % and 28.9 %, respectively. These results are very close to our estimations using the combined adjoint and MRIO analysis, in which the relative contributions from China and India from a consumption perspective are 18.4 % and 30.7 %, respectively. Therefore, we assume that the uncertainties related to the adjoint approach are within the range of 5~15 %. The uncertainties related to the meteorological conditions are considered in this study by conducting a multiyear adjoint analysis using the 2007-2011 meteorological data. As shown in Fig. 3c and Supplementary Fig. 4, the uncertainties of our estimated BC radiative forcing related to the climate variabilities are generally within the range of 10%, especially for major source regions including India, China and other Asian regions. In this study, we downscaled the 2° × 2.5° source-receptor

sensitivity map of BC over the HTP to a finer resolution using emission as a proxy, which assumes that the transport efficiency is the same within a model grid. Considering that the long-distance atmospheric transport of BC to the HTP glaciers are following the large-scale circulations in the atmosphere, the approach has negligible effects on the estimation of most countries/regions. As for the adjacent regions like Nepal, which are beneath the HTP, the sub-grid transport efficiency may be different due to the complex terrain effects. Hence, it may contribute to the uncertainties in estimations of these regions.

Large uncertainties may exist in the estimation of the BC radiative forcing over the HTP. Kopacz et al. [45] estimated that the BC DRF at five Tibetan glacial sites varies from 0.2 to 1.7 W m^{-2}. He at al. [46] derived a larger range of 0.7-4.3 W m^{-2} for the annual mean BC DRF over the whole plateau. Our result, which focuses on the HTP glacier regions, falls within the range of these previous estimations. The uncertainty in the estimated BC DRF is primarily contributed by errors in the modelled BC AAOD. While utilizing the same method as that employed herein, He et al.[46] estimated that the uncertainties in the BC DRF relying upon the GEOS-Chem-simulated BC AAOD were within a range from -75 to 80 %. Large uncertainties in the BC SAF are associated with inaccuracies in the estimated BC-induced snow albedo reduction. The calculation of the snow albedo reduction is closely dependent upon the snow grain shape, BC-snow mixing state, and BC coating state [47]. According to these various factors, the annual mean BC SAF over the HTP glaciers estimated in this study is 1.7-5.3 W m^{-2}, which means an uncertainty range from -48 to 60 %. Our result is consistent with the estimate of 1.5-5.0 W m^{-2} over the HTP from He et al. [46]. Additionally, dust deposited on the snow can lower snow albedo, which was not considered in this study to avoid additional uncertainty from dust simulations[48,49]. The Intergovernmental Panel on Climate Change (IPCC) Fifth Assessment Report indicated that co-existing dust may reduce BC SAF by approximately 20%[50]. In this study, the DRF and SAF of BC averaged over the HTP glacier region selected are further assigned to different regions and sectors according to their relative contributions to the total BC mass-burden in the receptor box. Here we assume that BC transported from different regions to the receptor box is equally important for the relevant radiative forcing. Given that in our study is confined to certain grids in which the glacier area are exceeds 2 % over the HTP, uncertainties related to this process are likely to be negligible.

Considering the computational intensity of running a large number of scenarios, a Monte Carlo simulation that integrates all of the uncertainties related to the emissions, the GEOS-Chem model and its adjoint, the RTM and the SSAM is prohibitive. The factors discussed above sometimes share common uncertainties that cannot be directly aggregated together. Therefore, it is difficult and far beyond the scope of this study to provide a combined uncertainty accounting for all potential sources of errors. In our analysis, most of these uncertainties are shared by all of the investigated regions and exhibit no effects on the estimated relative contributions from different regions to the BC radiative forcing over the HTP glaciers. The indirect radiative forcing of BC is not

considered here due to the lack of data and knowledge regarding BC-cloud interactions, which could cause much larger uncertainties than the effect of BC DRF [40]. Although such uncertainties and limitations are inevitable, this study provides valuable insights into the sharing of responsibilities among countries/regions with regard to the BC pollution over the HTP in addition to useful guidance for effective climate mitigation endeavours.

Supplementary References:

1 Wang, R. *et al.* Exposure to ambient black carbon derived from a unique inventory and high-resolution model. *Proceedings of the National Academy of Sciences* **111**, 2459-2463 (2014).

2 Van der Werf, G. R. *et al.* Global fire emissions and the contribution of deforestation, savanna, forest, agricultural, and peat fires (1997–2009). *Atmospheric Chemistry and Physics* **10**, 11707-11735 (2010).

3 Bond, T. C. *et al.* Historical emissions of black and organic carbon aerosol from energy-related combustion, 1850–2000. *Global Biogeochemical Cycles* **21**, GB2018 (2007).

4 Lu, Z., Zhang, Q. & Streets, D. G. Sulfur dioxide and primary carbonaceous aerosol emissions in China and India, 1996–2010. *Atmospheric Chemistry and Physics* **11**, 9839-9864 (2011).

5 Zhang, Q. *et al.* Asian emissions in 2006 for the NASA INTEX-B mission. *Atmospheric Chemistry and Physics* **9**, 5131-5153 (2009).

6 Kurokawa, J. *et al.* Emissions of air pollutants and greenhouse gases over Asian regions during 2000–2008: Regional Emission inventory in ASia (REAS) version 2. *Atmospheric Chemistry and Physics* **13**, 11019-11058 (2013).

7 He, C. *et al.* A global 3-D CTM evaluation of black carbon in the Tibetan Plateau. *Atmospheric Chemistry and Physics* **14**, 7091-7112 (2014).

8 Shen, Z. *et al.* Analysis of transpacific transport of black carbon during HIPPO-3: implications for black carbon aging. *Atmospheric Chemistry and Physics* **14**, 6315-6327 (2014).

9 Zhang, J., Liu, J., Tao, S. & Ban-Weiss, G. Long-range transport of black carbon to the Pacific Ocean and its dependence on aging timescale. *Atmospheric Chemistry and Physics* **15**, 11521-11535 (2015).

10 Park, R. J. *et al.* Export efficiency of black carbon aerosol in continental outflow: Global implications. *Journal of Geophysical Research: Atmospheres* **110**, D11205 (2005).

11 He, C. *et al.* Microphysics-based black carbon aging in a global CTM: constraints from HIPPO observations and implications for global black carbon budget. *Atmos. Chem. Phys.* **16**, 3077-3098 (2016).

12 Nair, V. S. *et al.* Simulation of South Asian aerosols for regional climate studies. *Journal of Geophysical Research: Atmospheres* **117**, D4 (2012).

13 Ram, K., Sarin, M. M. & Hegde, P. Long-term record of aerosol optical properties and chemical composition from a high-altitude site (Manora Peak) in Central Himalaya. *Atmospheric Chemistry and Physics* **10**, 11791-11803 (2010).

14 Ram, K., Sarin, M. M. & Tripathi, S. N. A 1 year record of carbonaceous aerosols from an urban site in the Indo-Gangetic Plain: Characterization, sources, and temporal variability. *Journal of Geophysical Research: Atmospheres* **115**, D24313 (2010).

15 Ganguly, D. *et al.* Retrieving the composition and concentration of aerosols over the Indo-Gangetic basin using CALIOP and AERONET data. *Geophysical Research Letters* **36**, L13806 (2009).

16 Carrico, C. M. *et al.* The importance of carbon and mineral dust to seasonal aerosol properties in the Nepal Himalaya. *Atmospheric Environment* **37**, 2811-2824 (2003).

17 Ming, J., Xiao, C., Sun, J., Kang, S. & Bonasoni, P. Carbonaceous particles in the atmosphere and precipitation of the Nam Co region, central Tibet. *Journal of Environmental Sciences* **22**, 1748-1756 (2010).

18 Qu, W. J. *et al.* Chemical composition of the background aerosol at two sites in southwestern

and northwestern China: potential influences of regional transport. *Tellus B* **60**, 657-673 (2008).

19 Babu, S. S. *et al.* High altitude (~ 4520 m amsl) measurements of black carbon aerosols over western trans‐Himalayas: Seasonal heterogeneity and source apportionment. *Journal of Geophysical Research: Atmospheres* **116**, D24201 (2011).

20 Cao, J.-J. *et al.* Concentrations, seasonal variations, and transport of carbonaceous aerosols at a remote Mountainous region in western China. *Atmospheric Environment* **43**, 4444-4452 (2009).

21 Zhao, Z. *et al.* Aerosol particles at a high‐altitude site on the Southeast Tibetan Plateau, China: implications for pollution transport from South Asia. *Journal of Geophysical Research: Atmospheres* **118**, 11360– 11375 (2013).

22 Beegum, S. N. *et al.* Spatial distribution of aerosol black carbon over India during pre-monsoon season. *Atmospheric Environment* **43**, 1071-1078 (2009).

23 Chen, X. T., Kang, S. C., Cong, Z. Y., Yang, J. H. & Ma, Y. M. Concentration, temporal variation, and sources of black carbon in the Mt. Everest region retrieved by real-time observation and simulation. *Atmospheric Chemistry and Physics* **18**, 12859-12875 (2018).

24 Wan, X. *et al.* Size distribution of carbonaceous aerosols at a high-altitude site on the central Tibetan Plateau (Nam Co Station, 4730ma.s.l.). *Atmospheric Research* **153**, 155-164 (2015).

25 Cong, Z. *et al.* Carbonaceous aerosols on the south edge of the Tibetan Plateau: concentrations, seasonality and sources. *Atmos. Chem. Phys.* **15**, 1573-1584 (2015).

26 Wang, M. *et al.* Two distinct patterns of seasonal variation of airborne black carbon over Tibetan Plateau. *Sci Total Environ* **573**, 1041-1052 (2016).

27 Zhao, S., Ming, J., Xiao, C., Sun, W. & Qin, X. A preliminary study on measurements of black carbon in the atmosphere of northwest Qilian Shan. *Journal of Environmental Sciences* **24**, 152-159 (2012).

28 Xu, B. *et al.* Black soot and the survival of Tibetan glaciers. *Proceedings of the National Academy of Sciences* **106**, 22114-22118 (2009).

29 Ming, J. *et al.* Black Carbon (BC) in the snow of glaciers in west China and its potential effects on albedos. *Atmospheric Research* **92**, 114-123 (2009).

30 Ming, J., Xiao, C., Du, Z. & Yang, X. An overview of black carbon deposition in High Asia glaciers and its impacts on radiation balance. *Advances in Water Resources* **55**, 80-87 (2013).

31 Ming, J., Du, Z., Xiao, C., Xu, X. & Zhang, D. Darkening of the mid-Himalaya glaciers since 2000 and the potential causes. *Environmental Research Letters* **7**, 014021 (2012).

32 Xu, B., Yao, T., Liu, X. & Wang, N. Elemental and organic carbon measurements with a two-step heating–gas chromatography system in snow samples from the Tibetan Plateau. *Annals of Glaciology* **43**, 257-262 (2006).

33 Ming, J., Xiao, C., Du, Z. & Flanner, M. Black Carbon in snow/ice of west China and its radiave forcing. *Advances in Climate Change Research* **92**, 114-123 (2009).

34 Li, X. F. *et al.* Light-absorbing impurities accelerate glacier melt in the Central Tibetan Plateau. *Sci Total Environ* **587**, 482-490 (2017).

35 Qu, B. *et al.* The decreasing albedo of the Zhadang glacier on western Nyainqentanglha and the role of light-absorbing impurities. *Atmos. Chem. Phys.* **14**, 11117-11128 (2014).

36 Zhang, Y. L. *et al.* Light-absorbing impurities enhance glacier albedo reduction in the southeastern Tibetan plateau. *J Geophys Res-Atmos* **122**, 6915-6933 (2017).

37 Yang, S., Xu, B., Cao, J., Zender, C. S. & Wang, M. Climate effect of black carbon aerosol in a Tibetan Plateau glacier. *Atmospheric Environment* **111**, 71-78 (2015).

38 Ming, J. *et al.* Grey Tienshan Urumqi Glacier No.1 and light-absorbing impurities. **23**, 9549-9558 (2016).

39 Xu, B. Q. *et al.* Post-depositional enrichment of black soot in snow-pack and accelerated melting of Tibetan glaciers. *Environmental Research Letters* **7**, 014022 (2012).

40 Bond, T. C. *et al.* Bounding the role of black carbon in the climate system: A scientific assessment. *Journal of Geophysical Research: Atmospheres* **118**, 5380-5552 (2013).

41 Peters, G. P., Minx, J. C., Weber, C. L. & Edenhofer, O. Growth in emission transfers via international trade from 1990 to 2008. *Proceedings of the national academy of sciences* **108**, 8903-8908 (2011).

42 Zhang, Q. *et al.* Transboundary health impacts of transported global air pollution and international trade. *Nature* **543**, 705-709 (2017).

43 Lin, J. *et al.* Global climate forcing of aerosols embodied in international trade. *Nature Geoscience* **9**, 790-794 (2016).

44 Qi, L., Li, Q., Henze, D. K., Tseng, H. L. & He, C. Sources of springtime surface black carbon in the Arctic: an adjoint analysis for April 2008. *Atmos. Chem. Phys.* **17**, 9697-9716 (2017).

45 Kopacz, M. *et al.* Origin and radiative forcing of black carbon transported to the Himalayas and Tibetan Plateau. *Atmospheric Chemistry and Physics* **11**, 2837-2852 (2011).

46 He, C. *et al.* Black carbon radiative forcing over the Tibetan Plateau. *Geophysical Research Letters* **41**, 7806-7813 (2014).

47 He, C. *et al.* Impact of Snow Grain Shape and Black Carbon–Snow Internal Mixing on Snow Optical Properties: Parameterizations for Climate Models. *Journal of Climate* **30**, 10019-10036 (2017).

48 Ridley, D. A., Heald, C. L., Kok, J. F. & Zhao, C. An observationally constrained estimate of global dust aerosol optical depth. *Atmospheric Chemistry and Physics* **16**, 15097-15117 (2016).

49 Kok, J. F. A scaling theory for the size distribution of emitted dust aerosols suggests climate models underestimate the size of the global dust cycle. Proceedings of the National Academy of Sciences **108**, 1016-1021 (2011).

50 Myhre, G. et al. in Climate Change 2013: The Physical Science Basis (eds Stocker, T. F. et al.) Ch. 8 (IPCC, Cambridge Univ. Press, 2013).
