# Supplementary material for: The cascade of global trade to large climate forcing over the Tibetan Plateau glaciers
Source: Nat Commun. 2019 Jul 23;10:3281. doi: 10.1038/s41467-019-10876-9 (PMC6650455; doi:10.1038/s41467-019-10876-9)
Supplement: Supplementary file 3 — Description of Additional Supplementary Files [file 41467_2019_10876_MOESM3_ESM.pdf]

Description of Additional Supplementary Files

File Name: **Supplementary Data 1.**

Description: Definition of 140 regions in the Global Trade Analysis Project (GTAP) version 9 database.

File Name: **Supplementary Data 2.**

Description: Definition of 57 sectors in the Global Trade Analysis Project (GTAP) version 9 database and the aggregated 13 sectors.
